# Four-Factor Prothrombin Complex Concentrate Reduces Time to Procedure in Vitamin K Antagonist-Treated Patients Experiencing Gastrointestinal Bleeding: A Post Hoc Analysis of Two Randomized Controlled Trials

**DOI:** 10.1155/2017/8024356

**Published:** 2017-09-19

**Authors:** Majed A. Refaai, Truptesh H. Kothari, Shana Straub, Jacob Falcon, Ravi Sarode, Joshua N. Goldstein, Andres Brainsky, Laurel Omert, Martin L. Lee, Truman J. Milling

**Affiliations:** ^1^University of Rochester Medical Center, Rochester, NY, USA; ^2^Seton Dell Medical School Stroke Institute, Dell Children's Medical Center, University Medical Center at Brackenridge, Austin, TX, USA; ^3^University of Texas Southwestern Medical Center, Dallas, TX, USA; ^4^Massachusetts General Hospital, Boston, MA, USA; ^5^CSL Behring, King of Prussia, PA, USA; ^6^UCLA Fielding School of Public Health, Los Angeles, CA, USA

## Abstract

**Introduction:**

To investigate the impact of a 4-factor prothrombin complex concentrate (4F-PCC [Beriplex®/Kcentra®]) versus plasma on “time to procedure” in patients with acute/severe gastrointestinal bleeding requiring rapid vitamin K antagonist (VKA) reversal prior to invasive procedure.

**Methods:**

A post hoc analysis of two phase III trials of 4F-PCC versus plasma in patients with acute/severe gastrointestinal bleeding. The treatment arms were compared for study treatment volume, infusion times, and time from start of study treatment to procedure.

**Results:**

Analysis included 42 patients (plasma, *n* = 20; 4F-PCC, *n* = 22). Median (interquartile range) infusion time was significantly shorter for the 4F-PCC group than for the plasma group (16 [13, 26] min versus 210 [149, 393] min; *P* < 0.0001). Median infusion volumes were significantly smaller (103 [80, 130] mL versus 870 [748, 1001] mL; *P* < 0.0001) and median time from study treatment initiation to first procedure was significantly shorter in the 4F-PCC group than in the plasma group (17.5 [12.8, 22.8] versus 23.9 [18.5, 62.0] h; *P* = 0.037).

**Conclusions:**

In this analysis of patients with acute/severe gastrointestinal bleeding requiring urgent VKA reversal prior to an invasive procedure, 4F-PCC (compared with plasma) was associated with smaller infusion volumes, shorter infusion times, and reduced time to procedure.

## 1. Introduction

Anticoagulants are routinely prescribed for the treatment and prevention of thromboembolic events. However, acute bleeding events in patients treated with oral anticoagulants are common [1]. The reported annual incidence of bleeding in anticoagulated patients is 15–20% [2]; major bleeding complications occur with an incidence of 1.7–3.4% [3]. In the United States, bleeding events in patients anticoagulated with vitamin K antagonists (VKAs) account for more than 60,000 annual emergency room visits [4].

Gastrointestinal (GI) bleeding is the most common major bleeding complication of VKA therapy [5, 6]; in the recent results published from the Outcomes Registry for Better Informed Treatment of Atrial Fibrillation (ORBIT-AF), GI bleeds represented 38% of major bleeding events in patients receiving warfarin [7]. GI bleeding is three times more common in patients with an international normalized ratio (INR) >3 than in those with INR 2-3 [5]. VKA-treated patients who experience acute major bleeding require rapid VKA reversal via the restoration of vitamin K-dependent coagulation factors (VKDFs); this can be achieved by administering plasma or prothrombin complex concentrates (PCCs).

Though widely used, plasma has several disadvantages when used for VKA reversal, including time delays due to blood group typing and thawing of frozen plasma, the need for large volumes and the associated long infusion times to achieve the necessary factor levels, and increased risk of transfusion reactions, such as volume overload and transfusion-related acute lung injury [8, 9]. PCCs are lyophilized products that are administered in smaller volumes over shorter periods of time; they are either activated or nonactivated. Activated PCCs are indicated for treatment of hemophilia A or B with inhibitors. Nonactivated PCCs are either 3-factor (3F-PCC, containing significant quantities of factors II, IX, and X) or 4-factor (4F-PCC, containing factors II, IX, X, and clinically relevant amounts of factor VII [9, 10]); these were initially developed for use in people with a congenital deficiency in VKDFs when purified specific coagulation factor is not available [11–15], with some now also being used for prevention or treatment of bleeding associated with VKA treatment [12, 14, 15].

Two multinational, multicenter phase IIIb clinical trials compared 4F-PCC with plasma for urgent VKA reversal [16, 17]. 4F-PCC was found to be noninferior to plasma for effective hemostasis and superior to plasma for rapid INR reduction in the study of patients with acute major bleeding [17] and superior to plasma for both of these endpoints in the study of patients needing VKA reversal prior to an urgent surgery or invasive procedure [16]. GI (and other nonvisible) bleeding was the most common type of bleeding reported in the acute major bleeding study, accounting for over 60% of bleeding events [17]. GI bleeding also occurred in the study of patients needing VKA reversal prior to an urgent surgery or invasive procedure [16]. This post hoc analysis evaluates the subset of patients at two US sites who had GI bleeding in either of the trials.

## 2. Methods

### 2.1. Study Design

Full details of the design of the acute major bleeding study (NCT00708435) and the urgent surgical or invasive interventions study (NCT00803101) have been published [16, 17]. Patients of both studies were randomly assigned (1 : 1) to receive either 4F-PCC (Kcentra, Beriplex P/N, CSL Behring, Marburg, Germany) or plasma. Both studies were sponsored by CSL Behring and performed in accordance with local ethics regulations; written informed consent was obtained from or on behalf of all patients.

Using data from two US sites that were major recruiters in the phase IIIb trials (University of Rochester Medical Center [URMC], Rochester, New York, and the Seton Family of Hospitals [SFH], Austin, Texas), we performed a post hoc analysis of patients who had GI bleeding events, to investigate the impact of 4F-PCC versus plasma treatment on time to procedure.

### 2.2. Patients

Inclusion criteria for the original studies have been published previously [16, 17]. GI bleeds were experienced by 113/212 (53%) patients in the acute major bleeding study. As bleeding events were not an inclusion criterion for the surgery study, such events were not systematically reported by all sites in that study. Patients from two study sites (URMC and SFH) who experienced GI bleeding events were included in this post hoc analysis. Together, these sites enrolled 112 patients (30%) for the phase III studies. Chart review and data abstraction for this post hoc analysis were performed by site personnel without additional funding.

### 2.3. Treatment

As previously described, on day 1, each patient received the assigned study treatment dosed according to baseline INR and body weight (Table 1) [16, 17]. 4F-PCC was administered as a single intravenous dose, with a maximum infusion rate of 3 IU/kg per min (equivalent to 8.4 mL/min [18]). Plasma was infused intravenously with a study-protocol-recommended rate of 1 U per 30 min interval in the acute bleeding study [17] and as rapidly as possible and at the discretion of the treating clinical team in the urgent surgical procedure study [16]. All patients were to receive vitamin K: in the bleeding study, by slow intravenous infusion dosed according to 2008 American College of Chest Physicians guidelines (5–10 mg) [19] or local clinical practice if different; in the surgery study, 2–10 mg (either oral or IV) at the clinician's discretion.

### 2.4. Assessments

Full details of the assessments performed for this study have been previously described [16, 17]. The primary endpoint of both studies was hemostatic efficacy and the coprimary endpoint was rapid INR reduction (≤1.3 at 0.5 h after the end of study treatment infusion). In this post hoc analysis of patients experiencing GI bleeding events requiring surgical or invasive procedures, we assessed dose, volume, duration of infusion, type of procedure, and timings (from admission to initiation of study treatment and to the start of procedure and from initiation of study treatment to start of procedure and total hospital stay duration). Procedures performed in this cohort were endoscopic, including esophagogastroduodenoscopy [EGD], colonoscopy, or sigmoidoscopy; one small bowel follow-through was also performed. We also report safety outcomes for this cohort.

### 2.5. Statistical Analysis

Between-group differences for study treatment infusion volume, study treatment infusion time, time from admission to study treatment, time from admission to first procedure, and time from start of study treatment to first procedure were assessed by the Wilcoxon rank-sum test. Between-group differences in hemostatic efficacy and INR reduction were evaluated using the chi-squared test/Fisher's exact test, depending on the cell sizes in the corresponding contingency table. A two-tailed 5% significance level was used throughout. This post hoc analysis was not powered to support the comparisons performed, so we treated testing of data as descriptive rather than inferential.

Quantitative data were summarized using mean/median and standard deviation/interquartile range (IQR).

## 3. Results

### 3.1. Patients

The intention-to-treat (ITT) efficacy population of the acute bleeding study and the urgent surgery/procedure study comprised 202 and 168 patients, respectively; the same number of patients (*n* = 185) received 4F-PCC as received plasma [16, 17]. This post hoc analysis included a total of 42 patients from the two study sites who experienced GI bleeding events; of these, 22 (52%) patients received 4F-PCC and 20 (48%) received plasma. The 4F-PCC group included 20 (90.9%) patients from the acute major bleeding study and 2 (9.1%) from the urgent surgery/procedure study. The plasma group included 17 (85%) patients from the acute major bleeding study and 3 (15%) from the urgent surgery/procedure study. Baseline data and characteristics were broadly similar between treatment groups in this analysis (Table 2). Median baseline INR in the acute major bleeding study was 4.6 and 3.2 for the 4F-PCC and plasma groups, respectively, with corresponding values of 2.5 and 2.8 for these groups in the urgent surgery/procedure study.

### 3.2. Study Treatments

The majority of patients in this analysis received a 4F-PCC dose of 25 IU/kg (11/22, 50%) or a plasma dose of 10 mL/kg (11/20, 55%). The study treatment doses are described fully in Table 3.

**Figure 1:**
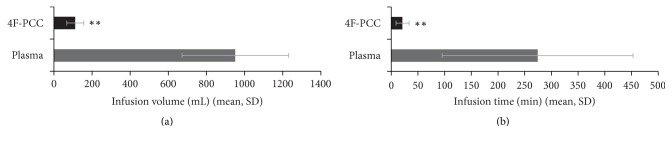
(a) Infusion volumes of study treatments. (b) Infusion times of study treatments. ^*∗∗*^*P* < 0.0001 compared with plasma. 4F-PCC, 4-factor prothrombin complex concentrate; min, minute; SD, standard deviation.

As shown in Figure 1(a), the mean study product infusion volume was approximately 8-fold smaller for patients receiving 4F-PCC than for those receiving plasma (*P* < 0.0001). Median (IQR) infusion volumes were 102.8 (80.0, 130.0) mL and 870.0 (747.5, 1000.5) mL for 4F-PCC and plasma, respectively.

The mean study treatment infusion time was approximately 13-fold shorter for the 4F-PCC group than the plasma group (*P* < 0.0001, Figure 1(b)). The median (IQR) infusion times were 16 (13, 26) min in the 4F-PCC group and 210 (149, 393) min in the plasma group.

### 3.3. Timing

**Figure 2:**
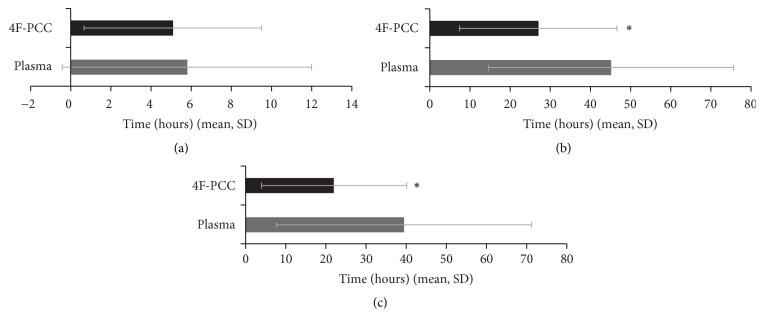
(a) Time between admission and start of study treatment. (b) Time between admission and first procedure. (c) Time between start of study treatment and first procedure. *P* value for comparison of groups for times from admission to study treatment = 0.64. ^*∗*^*P* < 0.05 compared with plasma. 4F-PCC, 4-factor prothrombin complex concentrate; SD, standard deviation.

The mean time between admission and start of study treatment was comparable for patients receiving 4F-PCC and plasma, as shown in Figure 2(a). The median (IQR) time from admission to initiation of study treatment was 4.0 (3.1, 5.9) h for 4F-PCC and 4.1 (3.2, 4.8) h for plasma (*P* = 0.64).

The mean time between hospital admission and first procedure was significantly shorter for the 4F-PCC group than the plasma group (*P* = 0.011), as shown in Figure 2(b). Median (IQR) times were 21.1 (16.4, 28.8) h for 4F-PCC and 29.7 (23.2, 66.3) h for plasma.

The mean time between start of study treatment and first procedure was also significantly shorter for the 4F-PCC group than the plasma group (*P* = 0.037), as shown in Figure 2(c). Median (IQR) times were 17.5 (12.8, 22.8) h for 4F-PCC and 23.9 (18.5, 62.0) h for plasma.

The median (IQR) total hospital stay duration was not different between treatment groups (6.0 [4.0, 10.5] and 5.0 [4.0, 8.0] days for 4F-PCC and plasma, resp.; *P* = 0.69). Mean (standard deviation) hospital stay duration (based on available data) was 8.1 (6.2) days in the 4F-PCC group (*n* = 20) and 7.7 (7.2) days in the plasma group (*n* = 19).

### 3.4. Surgical/Invasive Procedures

In this post hoc analysis, the urgent interventional procedures that patients underwent were all endoscopic procedures and included EGD, colonoscopy, and sigmoidoscopy. Full details regarding the types of procedures that patients underwent are provided in Supplementary Material available online at https://doi.org/10.1155/2017/8024356.

### 3.5. Nonstudy Blood Product Use

The numbers of patients who required nonstudy blood products, including packed red blood cells (PRBCs), and nonstudy plasma were comparable between the two groups. In the 4F-PCC group, 68% of patients (15/22) required PRBC transfusion, compared with 60% (12/20) in the plasma group. Two patients in the 4F-PCC group (9%) required nonstudy plasma compared with 3 (15%) from the plasma group.

### 3.6. Efficacy

Results for the primary and coprimary study endpoints of effective hemostasis and rapid INR reduction are described in Table 4. In the GI bleeds cohort, as in the original studies [16, 17], rapid INR reduction (≤1.3 at 0.5 h after infusion end) was observed more frequently in the 4F-PCC group than the plasma group (*P* < 0.0001). Furthermore, patients in the 4F-PCC group achieved INR ≤1.3 more rapidly after the start of infusion than those in the plasma group (*P* = 0.0001). Unlike the original studies, in the small GI bleeds cohort, there was no difference in hemostatic efficacy between groups.

### 3.7. Safety

Safety outcomes were consistent with the results from the two original multicenter clinical trials [16, 17, 23]. Adverse events (AEs) were reported for 15/22 patients (68%) in the 4F-PCC group and 17/20 patients (85%) in the plasma group. Serious AEs were reported for 4/20 patients (18%) in the 4F-PCC group and 7/22 patients (35%) in the plasma group. There was 1 fluid overload event in the 4F-PCC group (5%) and 4 in the plasma group (20%); no events in either group were deemed related to study treatment by the investigator. Thromboembolic events were experienced by 1 patient in the 4F-PCC group (5%) and 2 patients (10%) in the plasma group; one of the events in the plasma group was considered treatment-related. There were no deaths in either group. Safety results are tabulated in Supplementary Material (Additional File 2: Table S2).

## 4. Discussion

We found that time between start of infusion and start of procedure was significantly shorter in patients who received 4F-PCC compared with those in the plasma group. This reduced time to procedure was likely a consequence of the more rapid INR reduction and smaller infusion volume and therefore shorter infusion time with 4F-PCC versus plasma. The GI bleeds patient cohort mirrored the parent studies in that more of the 4F-PCC group than the plasma group met the coprimary endpoint of rapid INR reduction, demonstrating the efficacy of 4F-PCC. Though we did not see a similar effect on hemostatic efficacy, patient numbers in this study, as noted, were small.

It is important to establish whether a reduction in time to procedure is due to association or causation. Other factors which may affect time to procedure include hospital protocols guiding the initial management of patients with GI bleeding, the hemodynamic stability of the patient, and resource availability, including whether a 24-hour endoscopy service is available. However, as this analysis was performed using data from only 2 sites, variability in everything but individual patients should be limited.

Though in this small cohort we did not observe a significant difference in time between admission and start of study treatment between 4F-PCC- and plasma-treated patient groups, we speculate that a study of a larger sample would find a shorter time to treatment with 4F-PCC. This speculation is based on the established delays associated with plasma use, which requires blood typing and thawing [9], and partly due to sample size considerations. We note that our study reflects clinical trial practice rather than routine clinical practice, and as such all patients would have experienced minor delays associated with trial participation caused by review of inclusion/exclusion criteria, consent, and randomization to treatment. Therefore, in routine clinical practice, time to treatment with either plasma or 4F-PCC would likely be shorter than that found in this study. Though availability of thawed plasma in an emergency department reduces time to plasma infusion [24], we suggest that the larger infusion volume/longer infusion time with plasma versus 4F-PCC and the shorter time to reverse INR with 4F-PCC versus plasma will nonetheless consistently lead to an overall longer time to procedure with plasma versus 4F-PCC treatment in routine clinical practice.

### 4.1. Reducing Time to Procedure Could Improve Patient Outcomes

While the frequency of serious AEs and fluid overload was too small for meaningful statistical testing, our results appear consistent with the overall results of both trials. Plasma use is associated with an increased risk of fluid overload [9, 25]. This has been associated with increased hospital costs and increased length of hospital stay [25, 26]. In the setting of a study with a larger number of patients than the present analysis, this safety consideration could potentially contribute to reduced mean hospital stay in patients treated with 4F-PCC versus plasma. No relationship between frequency of fluid overload events and dose was noted for either plasma or 4F-PCC in the total study cohorts [23], and the low number of events (*n* = 5) in the population of patients with GI bleeds included here precludes any further specific analysis. Though there were no deaths in this cohort, mortality due to GI bleeding could potentially be reduced by expediting diagnosis and bleeding control with earlier diagnostic and therapeutic endoscopy. This is supported by a prospective analysis of patients with GI bleeding, which identified the lone independent risk factor associated with all-cause mortality in high risk patients (Glasgow-Blatchford Score ≥ 12) to be the time lapse between presentation and endoscopy [22] (Table 5).

### 4.2. Resource Use in Patients with GI Bleeds

GI bleeding may require hospitalization, laboratory tests, blood product use, diagnostic workup, and interventional procedures, potentially including surgery. A systematic review of patients with upper and lower GI bleeding demonstrated substantial healthcare costs associated with these conditions [27]. In this analysis, fewer patients received PRBC transfusions in the plasma group than the 4F-PCC group; however, given our small sample size, conclusive evidence for this should come from larger, future investigations.

In patients with GI bleeding, cost benefits may be expected from reducing hospital length of stay and the need for additional treatments or procedures (such as blood transfusions and further endoscopic or surgical intervention). However, the available data regarding cost benefits associated with early intervention in GI bleeding are conflicting [20–22] (Table 5). In the present analysis, reduced time to procedure was not associated with shorter hospital stay. Potential explanations for this include small sample size, comorbidities, and social factors affecting discharge.

### 4.3. 4F-PCC Impact on Procedural Efficacy in Patients with GI Bleeds

Given the improvement in time to procedure with 4F-PCC, analyses of association between time to surgical or invasive procedures and the efficacy of such procedures (assessed via incidence of rebleeding) would be of interest. Though this was beyond the scope of our investigation, it is something that should be addressed by future work.

### 4.4. Strengths and Limitations

The key limitation of this study is, as mentioned, its small sample size, as only a subset of patients from two larger studies were included. As this was a post hoc analysis, results cannot be considered conclusive and should be viewed as hypothesis-generating. In this study we benefitted from being able to draw on data not just for 4F-PCC-treated patients but also for a plasma-treated control group, to provide a balanced analysis of the outcomes tested herein.

## 5. Conclusions

Overall, in this post hoc analysis in patients experiencing GI bleeding while being treated with VKA anticoagulants, leading to a requirement for urgent surgical or invasive procedures, 4F-PCC was associated with smaller infusion volumes, shorter infusion times, and shorter times from admission to procedure, compared with plasma.

## Supplementary Material

Table S1: Corrective procedures that patients underwent, by center and treatment group.
Table S2: Summary of safety data.

## Figures and Tables

**Table 1:** Dose of study treatment per baseline INR.

Baseline INR	4F-PCC dose, IU of factor IX per kg body weight^a^	Plasma dose, mL per kg body weight^a^
2 to <4	25	10
4–6	35	12
>6	50	15

^a^Dose calculation based on 100 kg body weight for patients weighing >100 kg. Maximum dose ≤5000 IU of factor IX (4F-PCC) or ≤1500 mL (plasma). 4F-PCC, four-factor prothrombin complex concentrate; INR, international normalized ratio.

**Table 2:** Patient demographics and baseline disease characteristics.

	Plasma (*n* = 20)	4F-PCC (*n* = 22)
Study site, *n* (%)		
SFH	13 (65)	11 (50)
URMC	7 (35)	11 (50)
Study, *n* (%)		
Acute bleeding study	17 (85)	20 (90.9)
Urgent surgery/procedure study	3 (15)	2 (9.1)
Age, years		
Mean (SD; range)	75 (11; 51, 92)	73 (14; 33, 96)
Female gender, *n* (%)	11 (55)	9 (41)
Body mass index, kg/m^2^		
Mean (SD)	29.9 (6.5)	30.1 (13.5)
Initial (admission) Hgb, g/dL		
Mean (SD)	8.6 (2.9)	8.5 (2.1)
Initial (admission) Hct, %		
Mean (SD)	26.5 (8.1)	25.8 (6.2)
Initial (admission) INR		
Mean (SD)	4.7 (3)	5.5 (3.3)
Time on VKA treatment prior to study entry, days		
*n*	10	16
Mean (SD)	1784 (1851)	1503 (1604)
VKA dose, mg		
Mean (SD)	4.9 (2)	4.1 (2.1)
Indication for VKA treatment, *n* (%)		
Atrial fibrillation	11 (55)	16 (72)
Cerebral ischemia	3 (15)	1 (4.5)
Cardiac failure congestive	1 (5)	0
Pulmonary embolism	1 (5)	2 (9.1)
Aortic valve replacement	1 (5)	1 (4.5)
Deep vein thrombosis	1 (5)	2 (9.1)
Sick sinus syndrome	1 (5)	0
Coronary artery disease	1 (5)	0
Site of GI bleed^a^, *n* (%)		
Upper GI	3 (10)	4 (18.2)
Lower GI	3 (15)	5 (22.7)
Unspecified/data unavailable	14 (75)	13 (59.1)

^a^Where bleeding site was given as “probably upper,” site of bleed was considered unspecified. 4F-PCC, 4-factor prothrombin complex concentrate; GI, gastrointestinal; Hct, hematocrit; Hgb, hemoglobin; INR, international normalized ratio; SD, standard deviation; SFH, Seton Family of Hospitals, Austin, Texas; VKA, vitamin K antagonist; URMC, University of Rochester Medical Center, Rochester, New York.

**Table 3:** Study treatment doses.

	Patients, *n* (%)
Plasma dose (*n* = 20)	
10 mL/kg	11 (55)
12 mL/kg	4 (20)
15 mL/kg	5 (25)
4F-PCC dose (*n* = 22)	
25 IU/kg	11 (50)
35 IU/kg	4 (18)
50 IU/kg	7 (32)

4F-PCC, 4-factor prothrombin complex concentrate.

**Table 4:** Efficacy endpoints from the original phase IIIb studies.

	4F-PCC (*n* = 22)	Plasma (*n* = 20)	*P* value
Primary endpoint			
Hemostatic efficacy^a^, *n* (%)			
Excellent	12 (55)	11 (55)	0.98
Good	4 (18)	4 (20)	
Poor/none	6 (27)	5 (2.05)	

Coprimary endpoint Rapid INR reduction^b^, *n* (%)	13 (65)	0	<0.0001

Secondary endpoint: time from start of infusion to INR reduction ≤1.3, hours, mean (SD)	2.6 (5.5)	14.3 (8.8)	0.0001

^a^In both studies, hemostatic efficacy was assessed by an independent Endpoint Adjudication Board: over a 24-hour period from the start of infusion (bleeding study) or from the start of infusion to the end of procedure (surgery study), as excellent, good, or poor/none. (Surgery study hemostatic efficacy was originally reported as a binary endpoint [effective or noneffective] as assessed from the start of infusion to the end of the procedure, and effective hemostasis was defined as intraoperative blood loss not exceeding predicted blood loss by 30% or 50 mL, normal or mildly abnormal hemostasis [surgeon assessed], and no administration of nonstudy coagulation products). ^b^≤1.3 at 30 min after end of infusion. 4F-PCC, 4-factor prothrombin complex concentrate; INR, international normalized ratio; min, minutes; SD, standard deviation.

**Table 5:** Analyses of benefits associated with early intervention in patients with GI bleeding.

Study	Study design	Outcome with early intervention		
		Cost benefit/improved health resource utilization	Reduced hospital LOS	Improved clinical outcomes
Lee et al., 1999 [20]	(i) Prospective RCT	Yes	Yes	No
	(ii) Patients admitted with nonvariceal upper GI bleeding were randomized to either			
	(a) control group (elective endoscopy within 1-2 days; *n* = 54)			
	(b) early endoscopy group (endoscopy within 1-2 h; *n* = 56)			

Bjorkman et al., 2004 [21]	(i) Prospective, randomized, blinded, multicenter trial	No	No	No
	(ii) Patients with nonvariceal upper GI bleeding were randomized to either			
	(a) elective endoscopy (within 48 h of initial evaluation; *n* = 46)			
	(b) urgent endoscopy (within 6 h of initial evaluation; *n* = 47)			

Lim et al., 2011 [22]	(i) Prospective single-center trial enrolled 934 patients with nonvariceal upper GI bleeding	N/A	N/A	Yes (reduced mortality in high-risk patients)
	(ii) Lone independent risk factor associated with all-cause mortality in high risk patients (GBS ≥ 12) was found to be the time lapse between presentation and endoscopy			
	(iii) This association was not replicated in low-risk patients (GBS < 12)			

GBS, Glasgow-Blatchford Score; GI, gastrointestinal; h, hours; LOS,length of stay; N/A, not applicable; RCT, randomized controlled trial.
