# Supplementary material for: Four-Factor Prothrombin Complex Concentrate Reduces Time to Procedure in Vitamin K Antagonist-Treated Patients Experiencing Gastrointestinal Bleeding: A Post Hoc Analysis of Two Randomized Controlled Trials
Source: Emerg Med Int. 2017 Sep 19;2017:8024356. doi: 10.1155/2017/8024356 (PMC5625753; doi:10.1155/2017/8024356)
Supplement: Supplementary file 1 — Table S1: Corrective procedures that patients underwent, by center and treatment group. Table S2: Summary of safety data. [file 8024356.f1.docx]

**Supplemental digital content**

**Additional file 1: Table S1.** Corrective procedures

| **Treatment group** | **EGD only** | **Sigmoidoscopy only** | **Colonoscopy only** | **EGD and colonoscopy** |
| --- | --- | --- | --- | --- |
| *University of Rochester Medical Center, Rochester, New York (n, %)* | | | | |
| 4F-PCC | 6 (33.3) | 0 (0) | 0 (0) | 5 (27.8) |
| Plasma | 4 (22.2) | 1 (5.6) | 0 (0) | 2 (11.1) |
| *Seton Family of Hospitals, Austin, Texas (n, %)* | | | | |
| 4F-PCC | 2 (8.3) | 1 (4.2) | 4 (16.7) | 4 (16.7) |
| Plasma | 5 (20.8) | 0 (0) | 1 (4.2) | 7 (29.2) |

*4F-PCC* 4-factor prothrombin complex concentrate, *EGD* esophagogastroduodenoscopy

**Additional File 2: Table S2.** Safety results

| **Summary of AEs (n, %)** | | |
| --- | --- | --- |
|  | **4F-PCC (n = 22)** | **Plasma (n = 20)** |
| AE  Related AE^a^ | 15 (68)  0 | 17 (85)  3 (15) |
| SAE  Related SAE^a^ | 4 (18)  0 | 7 (35)  1 (5)^b^ |
| Death | 0 | 0 |
| Thromboembolic events  Serious  Related^a^ | 1 (5)  0  0 | 2 (10)  1 (5)^c^  1 (5)^c^ |
| Fluid overload  Serious  Related^a^ | 1 (5)  0  0 | 4 (20)  3 (15)  0 |
| Late bleeding episodes^d^  Serious  Related^a^ | 1 (5)  1 (5)^e^  0 | 0  0  0 |

All data are number of patients (%). ^a^Defined as events that were related to study treatment according to the investigator. ^b^Event of respiratory failure. ^c^The event considered serious was a separate event from the event considered related. ^d^SAE of bleeding occurring between 24 hours and 10 days after the start of study product administration (bleeding study), or SAE of bleeding occurring after the end of surgery (i.e., at time of wound closure) and within 10 days of study product infusion. ^e^Recurrent bleeding event attributed to re-anticoagulation. *4F-PCC* 4-factor prothrombin complex concentrate, *AE* adverse event, *SAE* serious adverse event
